# “That makes me feel human”: a qualitative evaluation of the acceptability of an HIV differentiated care intervention for formerly incarcerated people re-entering community settings in South Africa

**DOI:** 10.1186/s12913-022-08469-2

**Published:** 2022-08-26

**Authors:** Yangxi An, Nasiphi Ntombela, Christopher J. Hoffmann, Tolulope Fashina, Tonderai Mabuto, Jill Owczarzak

**Affiliations:** 1 Johns Hopkins University, 1550 Orleans St, CRB II – 1M11, Baltimore, MD 21205 USA; 2 Aurum Institute, Johannesburg, South Africa; 3 Department of Medicine, Johns Hopkins University School of Medicine, Baltimore, USA; 4 Department of Health, Behavior, and Society, Johns Hopkins University Bloomberg School of Public Health, Baltimore, USA

**Keywords:** HIV, Prison, Care continuum, South Africa, Acceptability, Peer

## Abstract

**Background:**

Correctional settings in South Africa have disproportionately high rates of HIV infection; a large number of inmates living with HIV return to the community each year. The transition community adherence club (TCAC) intervention was a differentiated care delivery approach with structural and peer components designed to increase antiretroviral therapy (ART) adherence and HIV care engagement following release from incarceration. The objective of this study was to assess the acceptability of the TCAC intervention among HIV-infected community re-entrants to inform program revisions and future intervention designs.

**Methods:**

This was a qualitative study set within a randomized controlled trial (RCT) of the TCAC intervention in South Africa. We conducted semi-structured, in-depth interviews with 16 re-entrants living with HIV and assigned to the intervention arm. All interviews were audio-recorded, transcribed, translated, and de-identified. Transcripts were coded and analyzed using content analysis, and acceptability was assessed using the Theoretical Framework of Acceptability (TFA).

**Results:**

Overall, study participants reported that the TCAC intervention was acceptable. Development of supportive relationships between participants, non-judgmental attitudes from peer-facilitators, and perceived effectiveness of the intervention to support ART adherence and HIV care were noted as the most valued components. An altruistic desire to help other participants facing similar post-incarceration and HIV-related challenges was a key motivator for TCAC attendance. A lack of access to reliable transportation to intervention sites and clinic-based medication collection were described as burdens to program participation. Illicit drug use by other group members and negative social influences were also identified as potential barriers to optimal program engagement.

**Conclusion:**

The TCAC was a well-accepted model of differentiated care delivery among re-entrants living with HIV in South Africa. To further enhance intervention acceptability for future scale-ups, program revisions should address logistical barriers related to reaching TCAC sites and implementing ART distribution at TCAC group sessions.

## Background

South Africa has one of the highest incarceration rates in the world, estimated at 235 people per 100,000 [1]. The reported HIV prevalence in South African correctional settings ranges from 15.6 to 25.3% [2, 3] and between 2 and 3% of all HIV-infected men in South Africa pass through the corrections system annually [4].

Studies have demonstrated that inmates who are provided with appropriate care and access to treatment in correctional facilities are receptive to ART initiation [5–7]. However, many face care transition challenges upon release, which leads to low engagement in HIV care and poor ART adherence during re-entry [8–11]. A study among 351 HIV-positive re-entrants in South Africa found that only 34% of participants had no lapse in ART supply within 90 days of release [12]. A meta-analysis of linkage to care studies from the United States (U.S.) reported similar proportions of post-release linkage to care with a median of 36% [13]. Development of an effective intervention to reduce interruptions in ART and HIV care among re-entrants is critical to ending the HIV epidemic. While multiple approaches have been tested in the U.S. [14–17], context specific interventions are needed in other settings, such as South Africa.

Community adherence clubs (CACs) are a model of differentiated care delivery that has been implemented in South Africa [18–20] and elsewhere in sub-Saharan Africa [21, 22]. CAC groups meet monthly or bimonthly for medication distribution and health assessment by a lay health worker. *Transition* community adherence clubs (TCACs) tailor the CAC model to overcome care engagement barriers that re-entrants face during their transition from incarceration to the community, including confusion regarding where and when to receive care, long clinic queues, limited social capital, enacted stigma, substance use, and re-incarceration [23–25]. The TCACs were designed for a closed group to meet biweekly with a curriculum and facilitated discussion led by a trained peer-facilitator.

This study aimed to assess the acceptability of the TCAC among re-entrants enrolled in a clinical trial of a TCAC intervention. Acceptability is a critical construct to the implementation of healthcare interventions [26]. An intervention’s acceptability to both implementers and recipients is an important determinant of its effectiveness in yielding favorable clinical outcomes and patient experience [27, 28]. Higher acceptability has been shown to improve intervention engagement and clinical outcomes [29, 30] while lower acceptability has been associated with poor treatment adherence and low intervention uptake [31, 32]. We sought to describe the acceptability of the TCAC intervention using the Theoretical Framework of Acceptability to inform program revisions, program scale-up, and future intervention designs.

## Methods

### Program description: transition community adherence Club

This study was part of a larger pilot randomized controlled trial (RCT) of the TCAC intervention (ClinicalTrials.gov NCT03340428 13/11/2017) [33]. The TCAC was tailored to overcome care transition challenges particularly affecting HIV-positive re-entrants. It was informed by a model of key barriers to care transition developed from prior work and the Behavioral Model for Vulnerable Populations [12, 17, 34]. The intervention was specifically designed to improve retention in HIV care and ART adherence among re-entrants by addressing logistical barriers to care, long wait times at clinics, clinic-based enacted stigma toward HIV and incarceration, low social support, substance use, and joblessness. Participants were assigned to the geographically closest TCAC venue with 4–14 other members upon release from a correctional facility. TCAC sessions were held every 2 weeks for a total of 12 sessions. Each session lasted approximately 2 hours and included a peer-facilitated group discussion, an interactive curriculum involving life and economic skills, HIV and health, disclosure and stigma, and individualized employment assistance. Health screening and ART distribution were scheduled to occur at TCAC sessions every 2 months.

### Sampling

Between November 2019 and November 2020, we recruited 16 re-entrants living with HIV who participated in the RCT of the TCAC intervention in a Province [location masked for blind review] in South Africa. We sought to attain maximum variation by transition in care status, duration of ART, and age group through purposive sampling. All interview participants provided informed consent and received reimbursement for study visits. The study was approved by [Ethics Committees and Institutional Review Boards masked for blind review].

### Data collection

Interviews were conducted by a researcher in the participant’s preferred language(s) (Sepedi, isiZulu, isiXhosa, Setswana, or English). Participants were interviewed in private settings such as office spaces or their homes, according to their stated request. Interviews followed a semi-structured, in-depth interview guide was informed by the Theoretical Framework for Acceptability (TFA) and TFA constructs through questions about perceptions of the care model; interactions with intervention facilitators and other participants; social networks, housing stability, and sources of support; and experiences with HIV and other non-HIV service providers. Interviews were audio-recorded, and the researcher took notes during interviews.

### Data analysis

Audio recordings were transcribed verbatim and translated into English (as necessary) by professional transcriptionists and translators. Transcripts were de-identified, and a pseudonym was assigned to each participant. Final transcripts were uploaded to MAXQDA software for coding; content analysis was used to analyze the transcripts [35, 36]. Specifically, we examined acceptability using the TFA (Table 1) [26]. The TFA has been applied to various evidence-based interventions, including prison-hospital interventions and mental health promotion programs [37, 38]. It defines acceptability as a “multi-faceted construct that reflects the extent to which people delivering or receiving a health care intervention consider it to be appropriate, based on anticipated or experienced cognitive and emotional responses to the intervention” [26]. This framework includes seven component constructs: (1) affective attitude; (2) perceived effectiveness; (3) burden; (4) self-efficacy; (5) opportunity cost; (6) intervention coherence; and (7) ethicality. We used these constructs to guide our interpretation of findings. Analysis included deductive and inductive coding, which enabled researchers to explore both theory-driven concepts from existing literature (e.g., treatment control) and novel topics of concern that may emerge from unstructured discussion (e.g., education).

**Table 1:** Component constructs of the theoretical framework of acceptability

Theoretical Framework of Acceptability (TFA)	Definition
Affective attitude	How an individual feels about the intervention
Perceived effectiveness	The extent to which the intervention is perceived as likely to achieve its purpose
Burden	The perceived amount of effort that is required to participate in the intervention
Self-efficacy	The participant’s confidence that they can perform the behavior(s) required to participate in the intervention
Opportunity cost	The extent to which benefits, profits or values must be given up to engage in the intervention
Intervention coherence	The extent to which the participant understands the intervention and how it works
Ethicality	The extent to which the intervention has good fit with an individual’s value system

Table 1 was originally developed and published in open access from Sekhon et al. [26] BMC HSR publication [see References]

In open-coding, two reviewers read all interview transcripts and wrote memos. Next, they explored the transcripts for concepts provided by an a priori coding guide. The a priori codes included stigma, access to healthcare, social capital, socioeconomic stability, and TCAC evaluation. Under each a priori code, subcodes were created to define common domains [39]. For example, the subcode lessons learned was formed under the a priori code TCAC evaluation. Inductive codes that emerged from the data but not explicitly addressed by the coding guide included drugs, physical sickness, religion and spirituality, education, and housing. The two reviewers reached a consensus on codes, and they coded the transcripts using the preliminary coding scheme. Another researcher retrieved the coded segments and adapted the subcodes to the seven TFA constructs. For instance, the subcode “lessons learned” was organized under the construct “perceived effectiveness.” A matrix was generated for the TFA framework, and the coded segments were charted into the matrix. Two other researchers reviewed the TFA codes to ensure their suitability and came to a consensus after several reconciliation meetings. Next, participants’ responses were synthesized to explore their positive and negative experiences with the intervention and additional challenges. The results are organized by themes within each TFA construct.

## Results

We interviewed 16 TCAC participants. All participants were men who identified as Black/African. The TCAC arm of the pilot RCT trial included 110 men and 6 women; we did not succeed in recruiting female participants for this study on acceptability. The median age was 34.5 years (interquartile range [IQR]: 31.3, 37.5), the median number of TCAC sessions attended by in-depth interview participants was 8 sessions (IQR: 6, 11), the median duration of incarceration was 1.09 years (IQR: 0.56, 2.98), and the median duration of ART was 0.5 year (IQR: 0.18, 2.55) (Table 2).

**Table 2:** Participant characteristics

Characteristics	*n*
*N*	16
Age group, years	
22–35	9
> 35	7
Gender	
Male	16
Female	0
Ethnic group	
Black/African	16
Indian/Asian	0
White/European	0
Language used in interview	
Sepedi	6
isiZulu	5
isiXhosa	1
Setswana	1
English	3
Employment status	
Unemployed	10
Informal/Piece jobs	6
Duration of incarceration	
< 1 year	7
1–2 years	4
> 2 years	4
Unknown	1
HIV care linkage since corrections release	
Linked to care	15
Not linked to care	1
Duration of ART	
< 1 year	8
1–2 years	3
> 2 years	3
Unknown	2

We identified themes within each of the TFA constructs (affective attitude, perceived effectiveness, burden, self-efficacy, opportunity cost, intervention coherence, and ethicality), and illustrative quotes from the themes were selected to exemplify both dominant and atypical patterns of data (Table 3).

**Table 3:** Illustrative quotes

Theoretical Framework of Acceptability (TFA)	Illustrative Quotes	Participant
Affective attitude	“I regard [the group] as a family. There are some things that I do not tell the people at home … I look for advice and solutions from the TCAC group first.”	P4
	“The thing that made it [meeting with the facilitator] to be simple... [is that] we –we are on a same side. He was also in prison himself...”	P1
	“Knowing the pain of *nyaope*, I get annoyed when … somebody leaving their homes has smoked [*nyaope*] … The person will greet and sit on a chair and then you will see that this person is high.”	P10
Perceived effectiveness	“It [the group] helped me to be consistent with the time I take my treatment because I never used to take it at thesame time so they [participants and facilitators] told me that the treatment is like a circle … you must drink it at the same time every day...”	P1
	“I say forward we go with the groups. There was a lot I did not know but now I do know them... I know how to speak to someone in a good manner, and the person will be free [feel comfortable] as well.”	P5
	“I got what I expected to get [from the TCAC] because I no longer feel the way I used to feel about myself, like feeling bad about myself.”	P3
Burden	“It happened [that the facilitator] told me that if I have money problems, we can provide it or pick you up where you stay and go to Soshanguve, I then said those are the good news indeed.”	P9
	“I will have to be in the queue for a long time [to get my medication].”	P11
	“There was a time where there was no train – I have had some challenges [with going to the clinic].”	P5
Self-efficacy	“If we were told that there is a session tomorrow, I would cancel all my other plans to attend. I would give myself those two hours … I wanted to go hear for myself...”	P2
	“I was shy and a little afraid the first time [first time attending TCAC sessions] … but as I continued talking with them, I opened up to them, I did not have stress.”	P1
Opportunity cost	“For me [the group] was fine and every time I would go there it was good because I knew that it removed me from a lot of things. Even my hustle [piece job] stresses me at times because you get different people, others will just swear at you.”	P3
	“Sometimes I was feeling, as if was just a waste of time … but as time goes on, I realized that the group is assisting me with many things. I also get support that I should be patient and I will get a job.”	P7
	“What I have sacrificed [in order to participate in the program] is my time, ensuring that each time they [facilitators] call me I avail myself for them … I made sure that they could get hold of me and on time. I have never missed their calls.”	P2
Intervention coherence	“We are speaking about health... we are assisting one another with our goals... we update one another with our achievement... also transport money, it help us a lot... you can buy airtime and cosmetic.”	P7
	“I think it is because I had not yet understood what [TCAC] was all about … That was the main reason … It’s because of things like that, that made me think of not coming back...”	P15
Ethicality	“I no longer indulge myself in bad thing … That is what this group helped me … if I can live like this, I won’t bother any person and at home I won’t hurt them.”	P11
	“There is nothing more important than having someone care for your wellbeing … When you get to the group, you have someone who leaves their home and come to motivate you.”	P4

### Affective attitude

The construct of affective attitude is concerned with the participants’ feelings about taking part in the TCAC. Participants had overall positive reflections regarding the intervention. They reported that supportive relationships that developed between group members motivated them to continue to attend TCAC sessions. They spoke of valuing the non-judgemental attitudes from other members, which helped establish an environment in which they felt comfortable to express themselves. “I was blessed to have people like [the group members] … because number one respect is there,” said P8. Participants also explained that they could trust other members to reciprocate respect and support, allowing them to actively seek advice from the groups.

Another aspect of the TCAC that engendered a positive attitude was the members’ ability to relate to the facilitators. The peer-facilitator with a history of incarceration was regarded as someone who could relate to the participants’ situation and serve as an example of overcoming care transition challenges. As P12 said, “The person that we can connect with truly is [the peer-facilitator] … he experienced everything that we experienced. When you talk he knows what you are talking about.”

Some participants found fault with aspects of the group structure, including mixing people who were actively using drugs with those who were not using. They were frustrated with group members who smoked *nyaope* (an opioid) and felt that the drug interfered with the decision-making capacity and working memory of the person using the drug, disrupting group dynamics and the participants’ ability to accomplish structured tasks. One participant even suggested that “the ones on drugs should be written off” from attending the group sessions.

### Perceived effectiveness

The construct of perceived effectiveness is the extent to which participants perceived the intervention as achieving its purpose. The TCAC’s primary goal was to increase HIV care engagement and ART adherence. Participants reflected that discussions of ART, participant narratives of success, and lessons about adherence techniques helped them develop tools and skills to regularly take their medications. The TCAC’s health and adherence curriculum, which addressed knowledge gaps in treatment, was viewed as effective as well.

The intervention also sought to establish social support to improve care engagement and the re-entry process overall. Participants reported that the program helped build relationships within and beyond the TCAC by facilitating group interactions and enhancing their interpersonal skills. For some members, the group assisted them with learning how to assess and respond to social situations appropriately. For example, P4 shared his experience of receiving guidance from his peer-facilitator, who dissuaded him from addressing family conflicts with violence.

Even though all participants commented on ways that the TCAC provided support, one participant, P7, was skeptical of the persistence of the TCAC’s influence outside of the group sessions. Specifically, he referred to negative social influences outside of the TCAC that made it a challenge for him (and he suggested other re-entrants) to follow through on the program’s behavioral objectives.

### Burden

The construct of burden focuses on the perceived amount of effort required to engage in the TCAC. Overall, few participants described particular burdens of attending the TCAC meetings. Transportation to the TCAC venue was one concern, and participants had to weigh the benefits of the intervention with the challenges of finding transportation. The TCAC facilitators sought to assist with this barrier by driving participants to the sessions when possible.

Participants spoke of the burden associated with clinic-based medication collection. Alleviating this burden was a goal of the TCACs, but efforts were unevenly implemented. The planned intervention component to provide ART at TCAC sessions was not fully implemented partly due to COVID-19 disruptions. Consequently, some participants described having to wait in long queues at local clinics, and those without stable access to transportation also had challenges with physically reaching the clinics.

### Self-efficacy in participating in the program

Self-efficacy concerns confidence to perform the behaviors necessary to participate in an intervention. Overall, participants expressed confidence in their ability to attend TCAC sessions. In most instances, the primary motivator for regular attendance was their desire to personally take part in discussions. Nonetheless, late arrival at sessions was a problem among participants, even those who lived close to the TCAC sites. Individuals who were offered free transportation were not confident in their ability to attend on time either, citing late pickups as a reason for tardiness.

Almost all participants noted that they actively contributed to group discussions. Even though several individuals reported feeling anxious and nervous during their first session, they were able to socially integrate into their groups over the intervention. “The first time we met … I was scared,” reflected P15, “then as time went on … we developed a bond.”

### Opportunity cost

The construct of opportunity cost is the extent to which benefits, profits, or values must be given-up in order to participate in the TCAC. Participants spoke of anticipating opportunity costs associated with their engagement in the program, questioning whether the program would be a good use of their time. Many re-entrants prioritized other responsibilities such as working at multiple informal jobs or looking after children over the TCAC. Nonetheless, most participants who actively attended sessions reported that the program was a meaningful use of their time. While some individuals reflected that the TCAC provided a respite from their busy schedules, others spoke of valuing the emotional and informational support that the groups offered.

### Intervention coherence

Findings related to intervention coherence are concerned with participants’ comprehension of the TCAC and how it works. Most participants described understanding the TCAC’s primary purpose to increase engagement in HIV care. “The sessions we are attending are about drugs and taking medication … they tell us not to relapse, we should take our medication.” One participant who was hesitant to attend the TCAC sessions identified his lack of understanding of the program’s aims as the primary reason for his reluctance. He anticipated that he would have to talk about his HIV status at group sessions, but what he found was discussions that supported HIV care engagement without solely focusing on HIV.

### Ethicality

The construct of ethicality focuses on the extent to which an intervention is perceived to be a good fit with the participants’ value system. Participants in the TCAC wanted to support the intervention by providing help to other people (both TCAC and community members) who were experiencing HIV care disruptions, recidivism, and drug addiction. As P1 said, “If I have something I want to say [in the group], I must say it and not keep quiet because I can help someone who does not know what I know.” This desire to help others in the group encouraged participants to continue to attend sessions.

Moreover, participants described the ethic of care and respect that the TCAC fostered. In particular, they appreciated how TCAC members genuinely cared about each other’s welfare. As P15 summarized: “You guys [TCAC] support me in terms of finding out on how was I doing or coping - that is the sign of support to me and that is humanity. That makes me feel human, that others care about me on this earth.”

## Discussion

The TCAC intervention is a novel structural and behavioral intervention designed to improve HIV care engagement and ART adherence during community re-entry. This study used qualitative methods to investigate the TCAC’s acceptability among HIV-positive individuals re-entering the community from correctional facilities in a higher-burden, urbanized setting in South Africa. The TFA proved to be a useful framework for assessing acceptability and capturing dimensions of the intervention that can be targeted to improve future scale-ups [26].

In qualitatively assessing the TCAC’s acceptability to participants, we observed overall acceptability through all domains of the TFA. The development of supportive relationships among participants and trust towards peer-facilitators were notable areas that engendered positive affective attitudes and perceptions of intervention effectiveness. The strength of peer-based interventions in facilitating HIV-related behavior change has been previously reported in diverse vulnerable populations, including re-entrants [40–42].

A desire to help other group members facing similar post-incarceration and HIV-related challenges as a motivator for TCAC attendance was another prominent theme among interviewed participants. Studies on peer-led HIV interventions have primarily focused on ways through which altruism encourages peer-facilitators to continue their involvement in peer support [43–46]. In our study, participants also reported having altruistic feelings for other group members and suggested that their attendance at TCAC sessions was motivated by their desire to help others. Past studies on peer-led interventions do not provide consistent findings on the extent to which participants are inclined to advise and pass-on acquired skills to each other [47–49], and research that investigates how the TCAC may have encouraged such behavior will be useful for informing future interventions.

The key burden to intervention participation was physically reaching the TCAC sessions. This finding, despite attempts to locate TCAC sites at easily accessible venues, highlights the challenge of transportation in South Africa. Limited access to cost-friendly or reliable transportation has been identified by studies to be a major barrier to intervention uptake and completion, especially those that require consistent, periodic attendance [50–52]. A future scale-up of the TCAC must consider the need for coordinated transportation to ensure program outcome and sustainability [53, 54].

Limited research has been completed on the acceptability of care transition interventions. Nonetheless, many studies on the perceptions of community-based adherence clubs in sub-Saharan Africa reported high acceptability among groups including post-partum women and health facility staff [21, 55, 56]. Consistent with our findings, participants in these studies cited supportive relationships and educational attainment as notable benefits of the clubs. One study from South Africa reported that although CACs were highly acceptable among PLWH, patients may have preferred clinic-based clubs over community-based clubs [19]. Loss from care was 38% higher in community-based clubs, and participants attributed this difference to easier access to healthcare providers at clinic-based clubs and potential stigma against HIV status within their communities. In contrast, TCAC participants discussed extra burden associated with clinic-based care and did not express concerns about the confidentiality of their HIV status.

Consistent with previous research on the acceptability of re-entry interventions, the TCAC’s life skills curriculum was deemed highly acceptable by participants [57, 58]. Life skills training may have been especially valuable for HIV-positive re-entrants as constructive relationships provide critical support that encourages linkage to care and mitigates medication non-adherence [23].

This study has the strength of using semi-structured interviews to assess acceptability, which allowed researchers to simultaneously examine themes guided by an interview protocol and collect new, exploratory data with follow-up questions [59]. This approach encouraged participants to share their perspectives on personal beliefs that underlay their behavioral patterns, which is crucial to understanding medication adherence [60]. Second, the analysis of the study’s results using a theoretical framework helped organize data in a systematic way that illustrates the relationship between variables [61]. Our findings can be useful in informing future interventions of variables that are relevant to achieving desired outcomes [62]. Lastly, the validity of interview data was established through the development of a coding system, peer briefing in data analysis, and memos that clarify researcher bias [63].

A potential limitation of this study is the limited range of participant experiences from which the data was obtained. While researchers attempted to attain a maximum variation sample, it is possible that certain interviewees were not selected using purposive sampling [64]. The COVID-19 pandemic may have prevented individuals from participating in the intervention, reducing the pool from which cases could be drawn. Some participants who were selected for interviews were also lost to follow-up, potentially obscuring varying viewpoints [65]. It should be noted that the planned TCAC component to distribute prepackaged medication to participants was not extensively implemented. This component intended to remove clinic-level barriers to care including long waiting times [66]. Most TCAC groups were unable to provide prepackaged ART to participants in the beginning due to logistic challenges, and ART distribution was later halted completely due to COVID-19 restrictions. Given that many participants discussed burden associated with clinic-based medication collection, it is likely that successful implementation of this planned component would be perceived positively. Furthermore, all participants in this study were men, so findings may only apply to male re-entrants. Previous research suggest that female re-entrants can have more specific health needs and experience greater levels of stigma and trauma than their male counterparts [67, 68]. This study was also conducted with a relatively small sample size in a single high-burden and urbanized region in South Africa. Its findings may not be generalizable to rural settings with a lower HIV prevalence or countries beyond South Africa.

The results of this study can be used to guide program revisions and inform future intervention designs. Our findings suggest that social support was a key contributing factor to high acceptability, and recruitment to promote TCACs should highlight the intervention’s intention and efficacy in fostering supportive relationships. Furthermore, program modifications can build on the highly acceptable peer-facilitation aspect of the intervention. Findings also point to areas for improvement, including a need to situate TCAC venues closer to participants’ places of residence and to overcome logistical challenges to medication distribution during TCAC sessions to eliminate the need to travel to clinics for routine HIV care. Future research is needed to examine (a) barriers to scaling up the TCAC; (b) whether participants with substance use disorders have needs that warrant the creation of separate TCAC groups that target recovery; (c) the TCAC’s acceptability among peer-facilitators and staff members; (d) how perceptions of the TCAC differ among participants who were incarcerated for different lengths of time; (e) how applied learning techniques can be integrated into the TCAC to improve re-entrants’ ability to transfer acquired skills to daily life; and (f) the extent to which altruistic intentions motivate program attendance among TCAC participants.

## Conclusion

The TCAC intervention, a group-based behavioral HIV care continuum intervention, was an acceptable model of differentiated care delivery among South African community re-entrants living with HIV. The acceptability of the TCAC was mediated by the development of supportive relationships among study participants, perceived effectiveness of the intervention in improving medication adherence and life skills, and altruistic intentions to help other people facing similar post-incarceration or HIV challenges. Program managers should build on the highly acceptable peer-facilitation aspect of the intervention in developing or implementing services for community re-entrants.

## Data Availability

The datasets used and/or analysed during the current study are available from the corresponding author on reasonable request. The qualitative datasets are not openly available in order to protect the participants’ privacy and confidentiality, particularly given the small sample size and the study’s geographic specificity. Study participants with stigmatized traits disclosed rich, detailed, and sensitive information that may unintentionally reveal their identities.

## Abbreviations

HIV: Human immunodeficiency virus
ART: Antiretroviral therapy
CAC: Community adherence club
TCAC: Transition community adherence club
TFA: Theoretical Framework of Acceptability
PLHIV: People living with HIV
